# Selectively inhibiting malignant melanoma migration and invasion in an engineered skin model using actin-targeting dinuclear Ru^II^-complexes†

**DOI:** 10.1039/d2md00280a

**Published:** 2022-11-01

**Authors:** Ahtasham Raza, Stuart A. Archer, Jim A. Thomas, Sheila MacNeil, John W. Haycock

**Affiliations:** a Materials Science & Engineering, University of Sheffield Mappin St Sheffield S1 3JD UK s.macneil@sheffield.ac.uk j.w.haycock@sheffield.ac.uk; b Department of Chemistry, University of Sheffield Brook Hill Sheffield S3 7HF UK james.thomas@sheffield.ac.uk +44 (0)114 222 9325

## Abstract

Due to the poor prognosis of metastatic cancers, there is a clinical need for agents with anti-metastatic activity. Here we report on the anti-metastatic effect of a previously reported Ru(ii) complex [{(phen)_2_Ru}_2_(tpphz)]^4+^, 1^4+^, that has recently been shown to disrupt actin fiber assembly. In this study, we investigated the anti-migratory effect of ^+^1^4+^ and a close structural analogue^+^, 2^4+^, on two highly invasive, metastatic human melanoma cell lines. Laser scanning confocal imaging was used to investigate the structure of actin filament and adhesion molecule vinculin and results show disassembly of central actin filaments and focal adhesions. The effect of both compounds on actin filaments was also found to be reversible. As these results revealed that the complexes were cytostatic and produced a significant inhibitory effect on the migration of both melanoma cell lines but not human dermal fibroblasts their effect on 3D-spheroids and a tissue-engineered living skin model were also investigated. These experiments demonstrated that the compounds inhibited the growth and invasiveness of the melanoma-based spheroidal tumor model and both complexes were found to penetrate the epidermis of the skin tissue model and inhibit the invasion of melanoma cells. Taken together, the cytostatic and antimigratory effects of the complexes results in an antimetastatic effect that totally prevent invasion of malignant melanoma into skin tissue.

## Abstract 2

A dinuclear ruthenium complex completely inhibits the invasive properties of malignant melanoma within a living human skin model.

## Graphical abstract

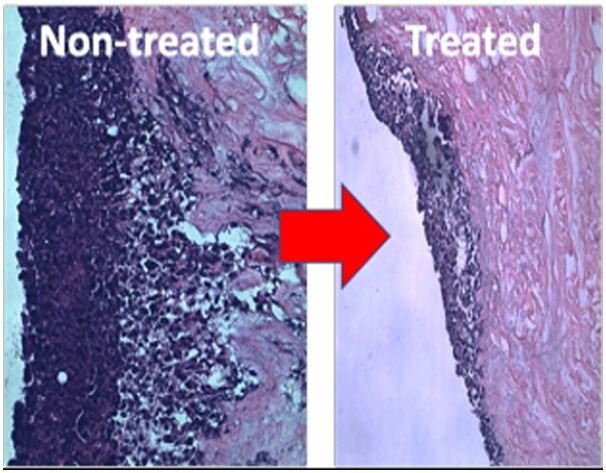

## Introduction

Over the decades following the success identification and application of cisplatin as an anticancer therapeutic,^1,2^ several related Pt^(II)^ complexes have moved into clinical application.^3–7^ Whilst the original SAR employed to identify these treatments has been exhausted,^8,9^ a number of Pt^II^ and Pt^IV^ leads unrelated to cisplatin are still being developed and^10–12^ complexes of other members of the platinum group metals have also been investigated;^13–16^ in particular, ruthenium compounds have attracted much attention.^17–20^

Although the original focus was on Ru^III^ systems,^21,22^ Ru^II^ complexes with promising therapeutic activity have also been identified. Predicated by the therapeutic action of cisplatin and its derivatives, the vast majority of these complexes have been designed to be classically cytotoxic.^23–29^ This approach has led to the identification and development of IT-139, a cytotoxic therapeutic lead that has been the subject of human trials.^30,31^

A small number of unconventional ruthenium leads have also been identified, perhaps the most well-known being NAMI-A.^32^ This compound is not cytotoxic nor is it significantly internalized into cells,^33–35^ yet it was the first ruthenium complex to enter clinical trials^36,37^ due to its *in vivo* anti-metastatic action.

More recently, research into the therapeutic action of kinetically inert ruthenium complexes, particularly polypyridyl Ru^II^ complexes, has rapidly evolved.^38–40^ By their nature, these complexes interact with biomolecules through reversible, non-covalent interactions, potentially leading to very different effects than cisplatin and analogues. They have also attracted attention because they possess photoactive excited states that can be exploited in applications such as imaging^41–44^ and phototherapeutics.^45–50^

In most of the associated therapeutic studies, it is assumed that such complexes interact with DNA and act as classical genotoxins or DNA-damaging photosensitizers. Comparatively less studies have investigated their interactions with proteins.

In pioneering work, the Meggers group carried out a program of studies on kinetically inert Ru^II^ complexes as protein kinase inhibitors.^51–54^ And more lately, the Martí group has shown that luminescent Ru^II^ complexes are effective probes for the formation of pathogenic protein fibrils.^55,56^

The possibility that this class of compounds could affect protein–protein interactions has also been explored. For example, a range of [Ru(bpy)_3_]^2+^ derivatives containing structurally complex ligands have been used to recognize extended surfaces of proteins such as cytochrome c and even inhibit the interaction between cytochrome c and cytochrome c peroxidase.^57–61^

**Fig. 1:**
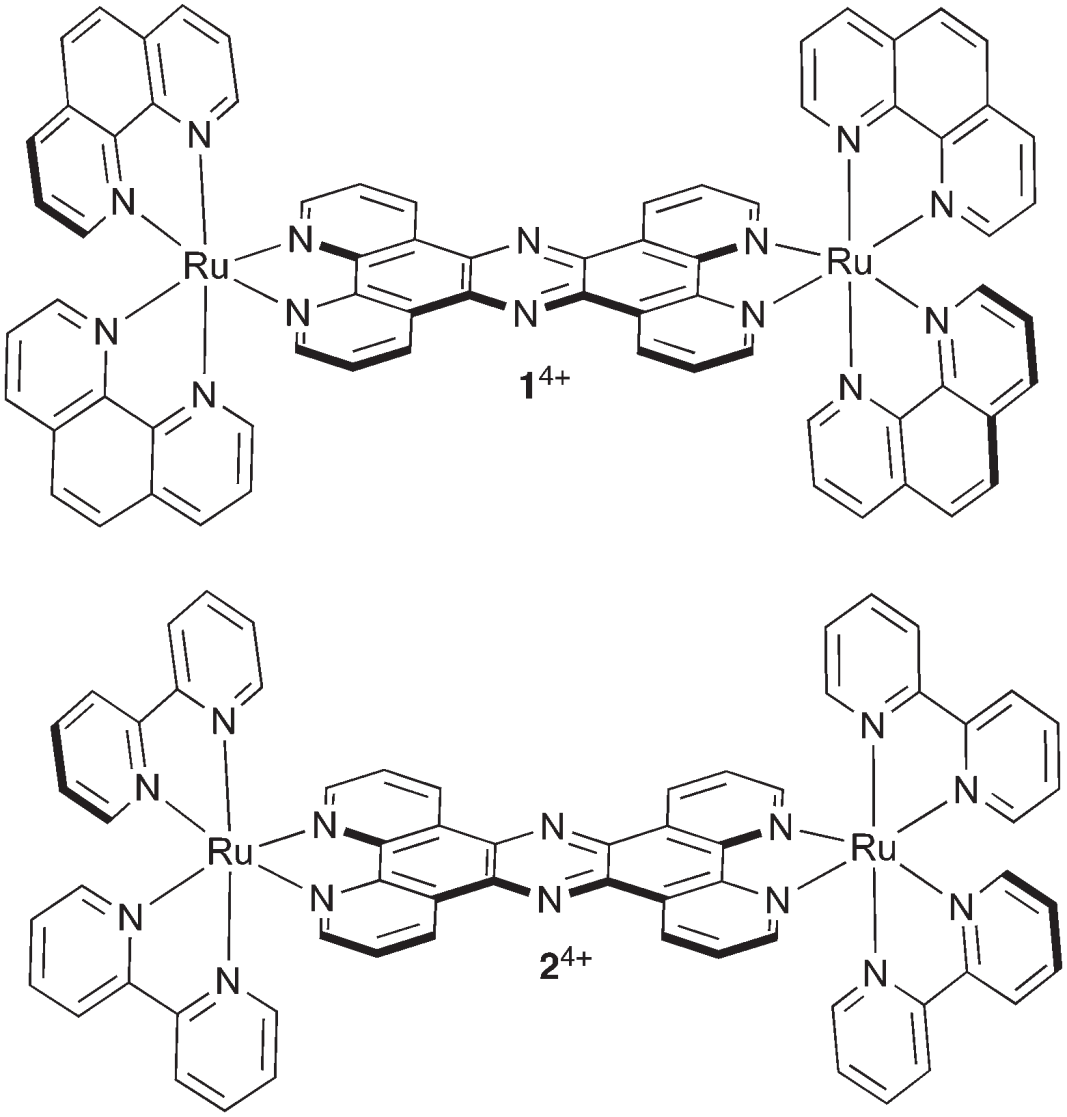
Structures of cationic complexes 1^4+^ and 2^4+^ studied in this report as their chloride salts.

Such effects can have interesting therapeutic implications; for example, recently, the MacDonnell group revealed that the mononuclear complex [Ru(DIP)_3_]^2+^ (DIP = 4,7-diphenyl-1,10-phenanthroline) promotes tubulin polymerization and stabilizes microtubules within cells through a high affinity binding interaction with assembled microtubules resulting in cellular effects that are comparable to paclitaxel.^62^ And in a very recent report we discussed the effect of a dinuclear Ru^II^ complex, 1^4+^, Fig. 1, on another cytoskeletal element, actin-based microfilaments.^63^

It was already established that this compound is neither classically genotoxic nor phototoxic;^42,43^ however, it was found that in live CP70 cells [1]Cl_4_ inhibits the interaction of G-actin subunits by binding to their surfaces, thus preventing their assembly into F-actin microfilaments, which leads to a late cytokinesis block and reduced cell motility.^63^ Ultimately, despite the absence of a DNA damage response or apoptosis signaling typically seen on treatment with genotoxic therapeutics, this interaction produces an inhibition of proliferation, an effect that has implications for the treatment of metastatic cancer.^64–66^

Metastases occur in later stages of tumorigenesis; when cancer cells leave a primary tumor and travel through the vascular and lymphatic system to distant sites in the body to form new colonies. This metastatic spread is closely associated with poor prognosis with up to 90% of cancer-related mortalities being associated with metastases^67,68^ and while the exact factors that lead to this process are not fully understood, they certainly involve degradation of the extracellular matrix (ECM), and increases in cellular migration, specific secondary seeding site, and angiogenesis.^69–71^ Indeed, the anti-metastatic effect of NAMI-A is thought to be caused by inhibition of angiogenesis and increasing adhesion, although how these effects occur is still not established.^32,72,73^ Therefore, the observed changes in cell motility and proliferation induced by 1^4+^ suggests that it offers potential as a novel anti-metastatic therapeutic.

Herein, we report the effect of 1^4+^ and its closely related analogue 2^4+^ on highly invasive and aggressively metastatic human malignant melanoma as represented by the two cell lines, A375-SM and C8161 and compare these data with those obtained with human dermal fibroblast, HDF, cells. In particular, the effect on melanoma cell adhesion and migration after short-term and long-term exposure to the two compounds was examined.

## Results and discussion

### Migration assay

**Fig. 2:**
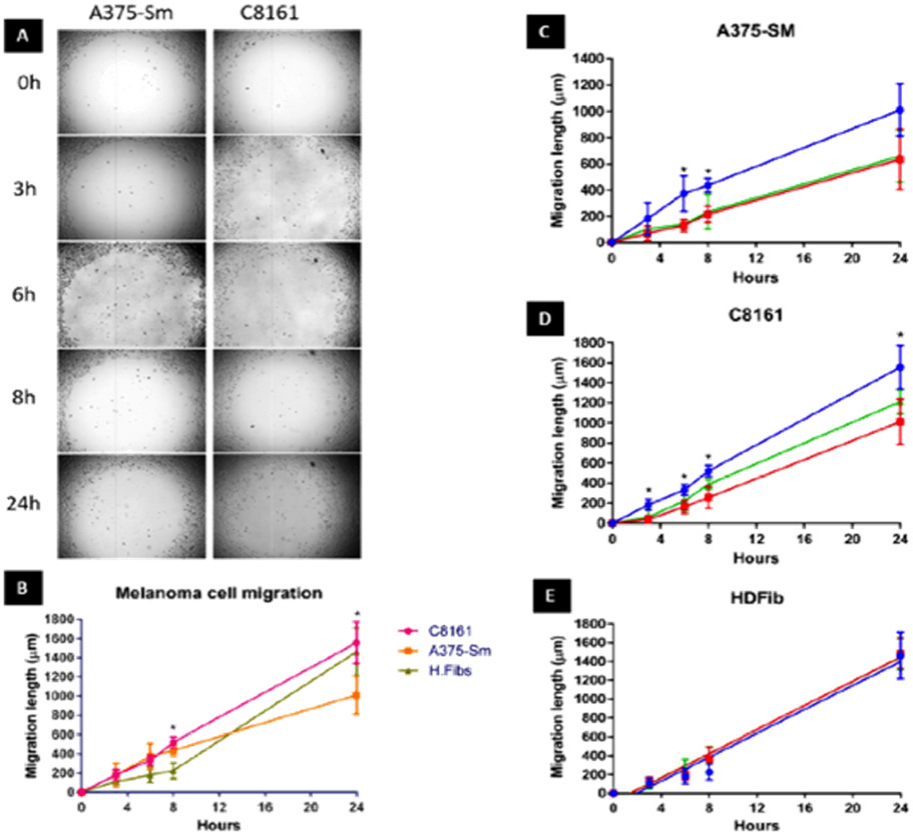
Human A375-SM and C8161 melanoma cell migration. (A) Investigation of two melanoma cell lines migration using cell exclusion zone created by cell stopper and cultured for 3,6,8 and 24 hours. (B) Comparison of distance migrated by melanoma cells and HDF over 24 hours. Inhibition of migratory distance by melanoma A375-SM (C), C8161 (D) and HDF (E) after 1 hour treatment with 1^4+^ (red line) or 2^4+^ (green line) compared to no treatment control (blue line) in serum free media.

We first set out to investigate what effect treatment with the complexes had on the mobility of melanoma cells using a standard scratch migration assay. The distance untreated cells migrated across a cell exclusion zone was measured at 0, 3, 6, 8 and 24 hours. Both A375-SM and C8161 cell lines rapidly migrated into the cell exclusion zone, though –over the 24 hours period– C8161 migration rates were faster than those of A375-SM and HDFs, (Fig. 2A and B). After cells were 90% confluent in a monolayer, a similar study was performed in serum-free media and rapid migration of melanoma cells was again evident (see ESI,† Fig. S1). We also confirmed our previous observation^74^ that these rates were predominately due to genuine migration processes rather than cell doubling times.

Remarkably, pre-incubation with the Ru(ii) compounds for one hour in serum free media prior to introduction of a cell exclusion zone resulted in a pronounced decrease in cell migration in both the melanoma cell lines (Fig. 2C and D).

A375-SM cell migration rates were most inhibited (∼40%), whilst C8161 cell migration rates were slightly less affected with inhibition rates of 25–32% being observed; a similar pattern of migration inhibition was seen in serum deprived melanoma cells (see ESI,† Fig. S1). Apart from the inhibitory effect on both A375-SM and C8161 melanoma cells, treatment with either complex has no detectable effect on the migration of HDF cells – Fig. 2E. If the metal complexes were first dissolved in 10% serum media their effect on melanoma cell migration was significantly inhibited, which is consistent with previous studies^75^ showing that this class of complex interacts with serum proteins, inhibiting their cellular uptake.

### Effect on actin filaments

To visualize actin filaments, both A375-SM and C8161 melanoma cell lines were labelled with phalloidin TRITC. To ensure that actin filaments were studied independent of interference from effects caused by mitosis, cells were starved overnight so that they were synchronized in their cell cycle.

Actin filaments are the most prominent feature in stress fibers, the bundles of filaments and myosin binding proteins that are crucial for cell contractility and mechano-sensing. It has been previously observed that motile cells contain thinner and more dynamic stress fibers^76,77^ and at enhanced magnification melanoma cell lines showed long and thin actin filaments along the axis of cell, with emission intensities being weaker in the center of the cells compared to cell cortices (see ESI,† Fig. S2).

**Fig. 3:**
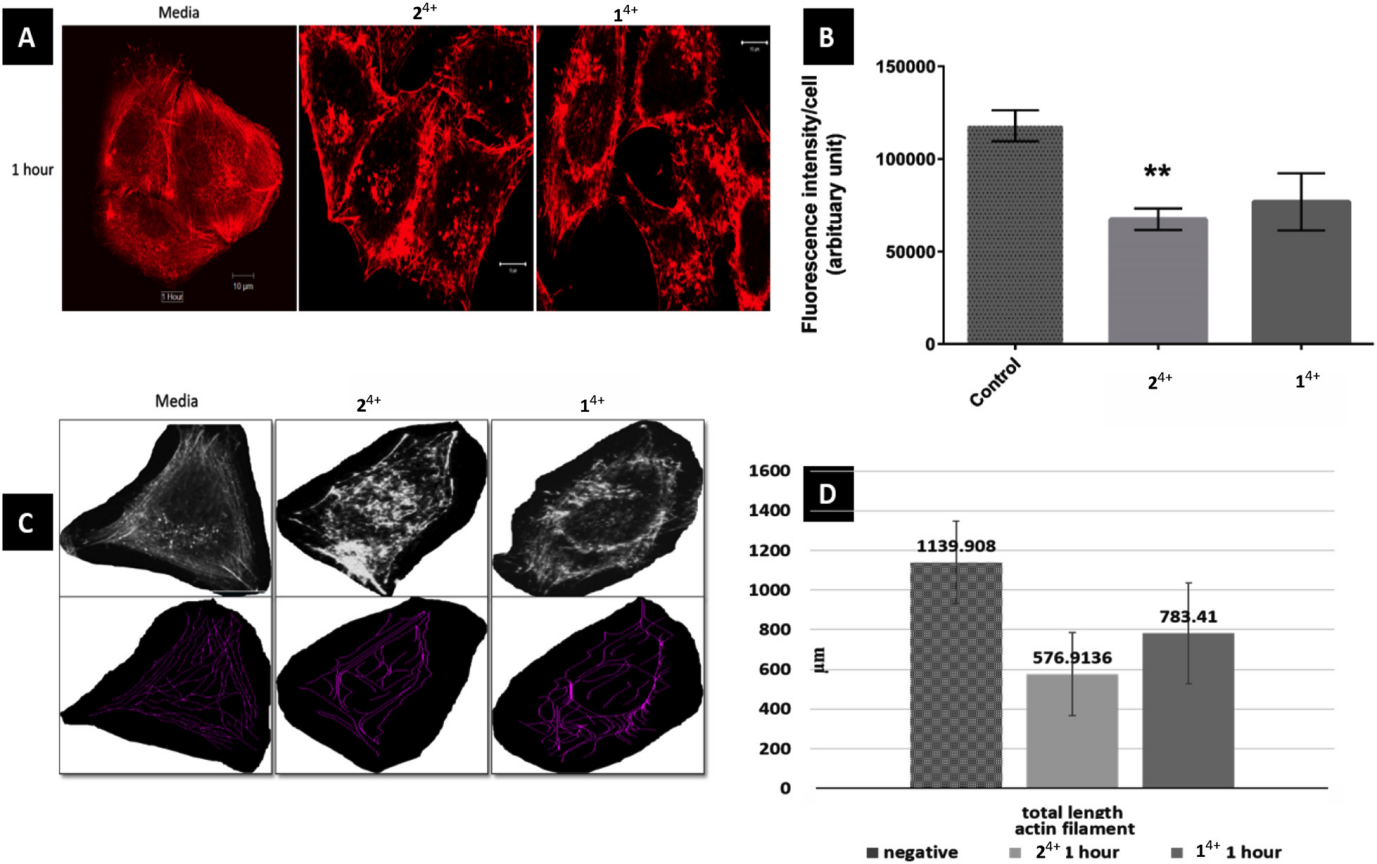
Effect of 1^4+^ and 2^4+^ on actin filaments of melanoma cell line A375-SM. (A) Actin filaments in cell center are more disruptive than outer filament just after 1 hour treatment of Ru-Phen and Ru-Bpy (magnification 40X, SB 10 μm) (B) corrected fluorescence intensity per cell was much reduced in Ru-Bpy than Ru-Phen as compared to normal group (*p* = 0.0013 and 0.051 respectively) (C) measurement of actin filaments using NeuronJ plug-in (D) total length of actin filaments has reduced significantly in treated group.

Initially, the short-term effects on A375-SM cells treated with either complex were investigated. They were exposed to the complexes for 1 hour in serum-free media and then fixed for phalloidin labelling, after which analysis was carried out through measurement of filament lengths within a single cell – Fig. 3. This revealed that the total length of actin filaments within these cells was significantly reduced, with the total actin filament length of the 2^4+^ treated group (576.91 ± 209 μm) being considerably shorter than the 1^4+^ treated group (783.41 ± 253.82 μm). However, both these measurements were both significantly shorter than the length measured in untreated cells (1139.9 ± 207.92 μm). Having observed this effect, a more detailed morphological experiment involving both melanoma cell lines treated with 1^4+^ and 2^4+^ for 1, 3, 6 and 24 hours was carried out.

**Fig. 4:**
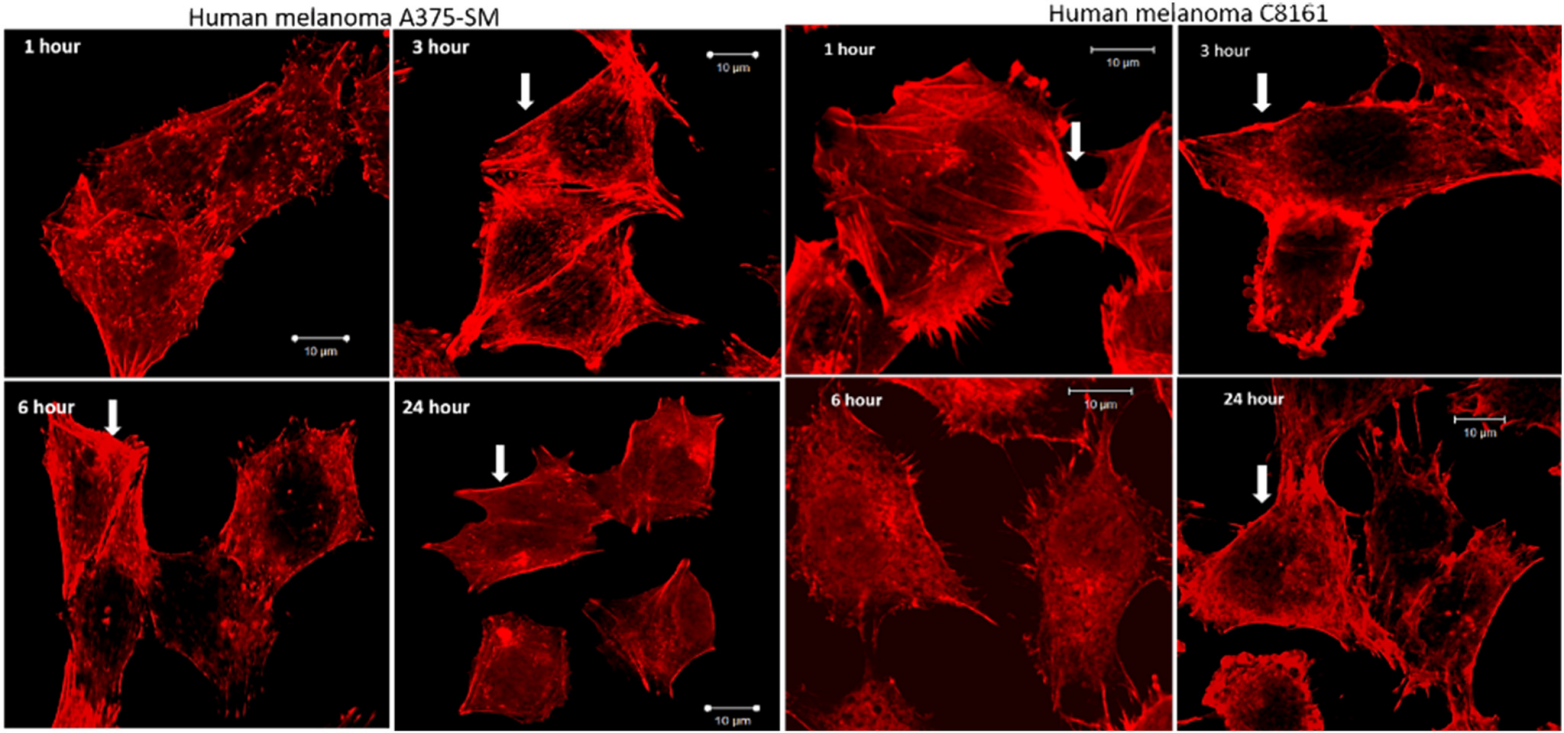
Effect of actin filaments after treatment with 2^4+^ for 1, 3, 6, and 24 hours. Progressive loss of central actin filaments while the cortical filaments (arrowhead) remain prominently in contact. A loss of cell size after 24 hour treatment is also visible (magnification 40X, SB 10 μm).

After treatment and phalloidin labeling, any differences in actin filaments morphology were analyzed. Fig. 4 shows a representative sequential series of images after treatment with 2^4+^; equivalent images for treatment with 1^4+^ are shown in the ESI,† Fig. S3.

**Fig. 5:**
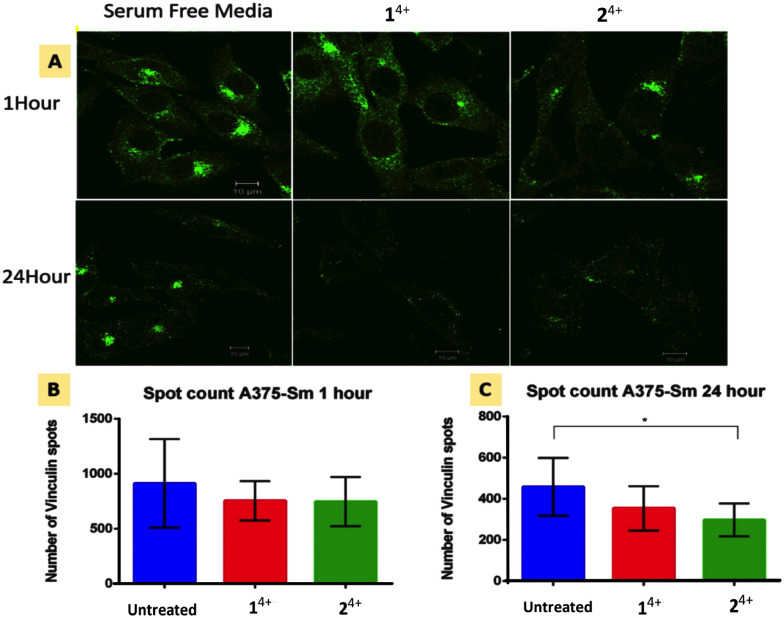
Focal adhesion response in human melanoma A375-SM cells after 1 and 24 hour treatment with Ru(ii) compounds. (A) Immunofluorescence imaging of vinculin spots with in cells. (B and C) Vinculin spot count after 1 hour and 24 hour treatment (*p* = 0.05, significant difference between no Rx and Ru–Ru Phe or Bpy group was 0.2 and 0.05 respectively).

While actin filaments in non-treated cells appeared long and undisrupted throughout the entire length of the cells, treated cells of both lines displayed a progressive loss of actin filaments over time, which was more prominent in central actin filaments than peripheral filaments (Fig. 5, peripheral actin filament arrowhead). Furthermore, although peripheral actin extensions (likely lamellipodia/filopodia/invadopodia) could be observed throughout the experiment, their overall numbers dropped considerably at later time intervals; a trend that was accompanied by a substantial loss of cell size.

When melanoma cells were treated with 1^4+^ or 2^4+^ for an hour then washed and replenished with fresh media, after six hours actin filaments started redeveloping, reaching almost complete recovery within 24 hours. Again, it was noticed that central actin filaments were particularly disrupted and took the longest to recover (see ESI,† Fig. S4). These observations, demonstrating the reversible effect of these compounds on actin filaments, further support our hypothesize that 1^4+^ disrupts the interaction of G-actin assembly through reversible non-covalent binding effects.^63^

### Vinculin disruption

Cell migration is driven by the interplay between F-actin and transmembrane integrin receptors; by binding to extracellular ligands, the external domains of integrin at focal adhesions provide the first step in the transduction of adhesion into movement; whilst the linkage of integrin internal domains to stress fibers provides a conduit to couple mechanical forces to the cytoskeleton.^78^ This relationship suggests that treatment with 1^4+^ or 2^4+^ will ultimately inhibit focal adhesion function. To explore this hypothesis, we investigated the effect of treatment with the complexes on the cytoskeletal protein vinculin which functions as a linking agent between integrin and the cytoskeleton, anchoring stress fibers to focal adhesions at the cell membrane.^79,80^

Immuno-fluorescent analysis of vinculin was performed on both melanoma lines. The cells were fixed after 1 and 24 hour treatment with either 1^4+^ or 2^4+^ and then labelled with rabbit monoclonal vinculin antibody. The imaging data was then analyzed to measure the vinculin spot count per cell; Fig. 5 shows the results of such an analysis for the A3375-SM line (see the ESI,† Fig. S5, for equivalent data for C8161 melanoma cells).

Whilst no effects were observed after 1 hour of treatment, after 24 hour both melanoma lines showed reductions in vinculin spot clusters. This suggests that the loosening of central actin filament observed after 1 hour treatment does not initially affect the firm anchoring of focal adhesions. After 24 hour treatment with either complex, stress fibers become so shortened and disarranged that they are no longer mechanical linked to focal adhesions leading to a concomitant drop in vinculin levels.

### Effects on cell viability

In our initial study, we found that that even though 1^4+^ is not genotoxic it has a potent cytostatic effect on breast and ovarian cancer cell lines as its interaction with actin ultimately disrupts late cytokinesis.^63^ As melanoma is notoriously resistant to most anti-neoplastic chemotherapeutics due to aberrant apoptotic signaling,^81^ we investigated the effect of 1^4+^ or 2^4+^ on both of aggressively invasive melanoma lines.

MTT assays showed a significant reduction in cell metabolic activity after 24 hour of treatment in both cell lines compared to no treatment (in serum free media) – see ESI.† For example, the cell metabolic activities after 24 hour of treatment with 1^4+^ (at 100 μM) in A375-SM and C8161 were reduced to 89% ± 19% and 65% ± 21% of their original activity respectively and were reduced to 89% ± 24 and 87% ± 29 on treatment with 2^4+^ at the same concentrations. At still higher concentrations more profound effects were observed: exposure to 200 μM of 1^4+^ resulted in a decrease in metabolic activity of 53% ± 13% in C8161 cells after 24 hours' treatment.

With the observation of both antimigratory and cytostatic effects in both melanoma lines we went on to investigate the effect of 1^4+^ or 2^4+^ in 3-D tumor and tissue models.

### Melanoma spheroid studies

Cell spheroids provide an excellent 3D preclinical model for studying therapeutic efficacy.^50^ As they provide more clinically relevant cellular conditions, they can help to reduce drug attrition rates in clinical trials. With this in mind, we used spheroids to investigate the effect of 1^4+^ and 2^4+^ on melanoma spheroids.

Using a previously described procedure, C8161 spheroids were cultured for three days until a consistent size (555 ± 50.5 μm) and quality (exponential growth) was achieved. Day 3 spheroid were then treated with 1^4+^ or 2^4+^ at concentrations of 10, 50, 100, 200 and 500 μM respectively in serum free media.

Exposure to 1^4+^ at the highest concentration of 500 μM produced a 15% reduction of spheroid growth (569 ± 20 to 493 ± 180), whereas treatment with 2^4+^ at the same concentration produced a ∼20% reduction in growth (569 ± 20 to 451 ± 40) over 7 days. The metabolic activity of the spheroids, tested on day 7 using MTT assays, also revealed that exposure to the complexes resulted in a viability decrease of ∼30% – see ESI,† Fig. S6.

Interestingly, this effect showed a threshold concentration: whilst the decrease in spheroid size and metabolic activity in response to a 200 μM treatment of 1^4+^ or 2^4+^ was also most identical to that at the higher dose, lower dose (10, 50, 100 μM) produced a much less pronounced effect. Having observed this distinctive effect on spheroid growth, we set out to investigate whether exposure to 1^4+^ and 2^4+^ altered the invasive properties of the C8161 spheroids.

**Fig. 6:**
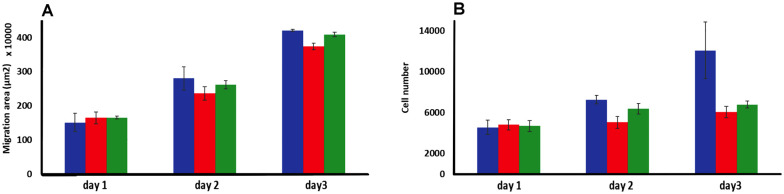
Invasion studies using C8161 melanoma spheroids embedding in a rat tail collagen 1 gel matrix. (A) Melanoma cells migrated area and (B) total number of migrated cells analyzed (over 3 days) after a bolus treating at day 1 with 500 μM of 1^4+^ (red) or 2^4+^ (green) compared to no treatment (blue) (*n* = 3).

Spheroids embedded in a suitable matrix also act as excellent physiological relevant 3D model for tumor invasion, as the matrix facilitates the cancer cells' ability to anchor, adhere, and migrate away from the primary spheroid site. In our migration studies a previous procedure^82^ was adapted to embed C8161 melanoma spheroids in rat tail collagen 1 gel which functioned as a model of the extracellular matrix, ECM. After the spheroids were exposed to a bolus dose of 1^4+^ or 2^4+^, the number of migrating cells and total migration area were then evaluated over the period of several days and compared to a nontreated control. This procedure resulted in some distinctive results – Fig. 6.

Although there was no significant difference in the extent of migration area between treated and untreated spheroids, the total number of cells migrating from the primary spheroid area was significantly reduced after treatment. Exposure to a single dose of either 1^4+^ or 2^4+^ led to a reduction in migrating cells of ∼30% after two days and 50% after three days compared to the untreated spheroids. These observations confirm that a bolus dose of either complex can successfully diffuse through the collagen ECM model and penetrate multicellular spheroids resulting in significant therapeutic effects.

The fact that there is a reduction in migrated tumor number from the primary area but not in the extent of spheroid migration distance is consistent with the previously discussed dose-dependent threshold effect observed in spheroid growth and suggests that the population of cells that do not receive an above-the-threshold dose of the complex are still migratory.

As both complexes affect cancer cell viability and the invasiveness of spheroid tumor model, a study to assess their efficacy in inhibiting melanoma invasion through a tissue-engineered human skin model was initiated.

### Inhibition of melanoma invasion through a human skin model

The use of tissue-engineered skin is particularly valuable in studies on invasive melanoma as the live skin model used in our experiments – first reported and extensively studied by the MacNeil lab – provides physiologically relevant dermal and epidermal structures, including a keratinized stratum corneum.^83,84^ For the invasion studies, isolated primary human keratinocytes, fibroblasts, and C8161 melanoma spheroids were submerged in media and co-cultured on an acellular skin dermis, then raised to an air–liquid interface, ALI, culture. A model of normal skin was developed through the same protocol, but without the addition of C8161 melanoma spheroids. After 14 days, both ALI cultures were treated with 1^4+^ or 2^4+^ and the results were compared to an untreated skin model.

Treatment of the normal skin tissue model with either complex led to observable changes in morphology; in particular, even at low treatment concentrations, some separation of the epidermal and dermal layers occurred due to disruption of the basement membrane of the dermal–epidermal junction. At higher concentrations a thickening and sloughing of the stratum corneum occurred; epidermal parakeratosis like this is seen in conditions such as psoriasis – see ESI,† Fig. S7.

When melanoma spheroids are incorporated within tissue engineered human skin, the biological architecture of this model very closely approximates that of native skin tissue suffering from melanoma metastasis. Indeed, as previously reported,^85^ the C8161 cell spheroids proved to be highly metastatic and rapidly showed extensive and aggressive invasion into the dermis of untreated tissues.

**Fig. 7:**
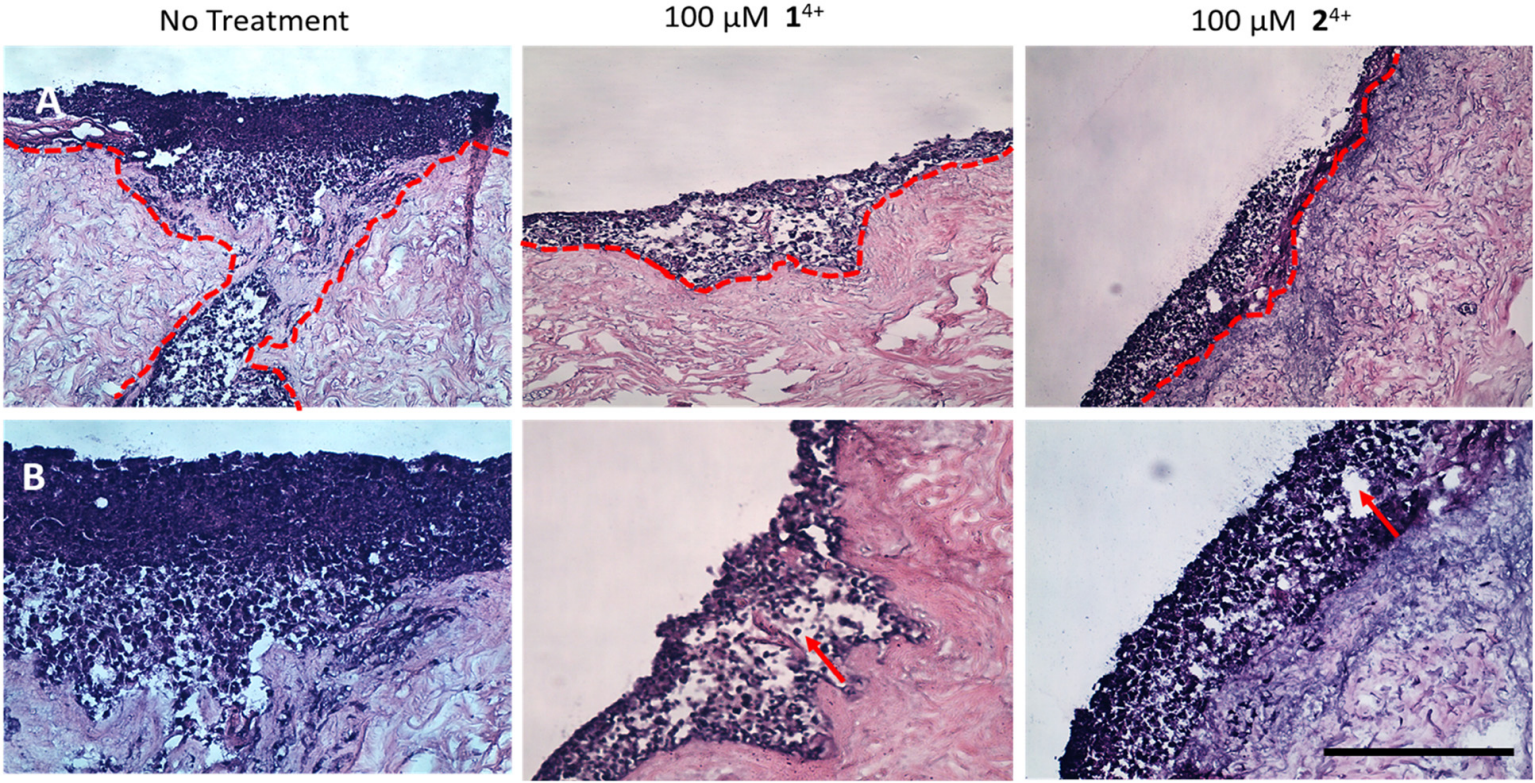
H&E-stained tissue engineered skin model. A. At ×10 magnification B. At ×20 magnification. The model was developed using melanoma spheroid (day 3) and primary cells (human isolated fibroblast and keratinocytes) cultured together on a human de-epithelialized dermis for 14 days (air–liquid interface culture conditions). The untreated control (left) was compared to the model dosed with either 1^4+^ (middle) or 2^4+^ (right). After 24 hours, the tissues were fixed and processed for histology. Melanoma cell migration from epidermis to dermis is observed in untreated tissues (red dotted line), while little to no cell migration was seen in the treated group. Vacuolar fluid-filled cells in the epidermis provide evidence of spongiosis. (red arrow) (SB = 500 μm).

In contrast with these observations, treatment with either complex resulted in minimal or even no invasion of melanoma cells into surrounding tissue, confirming the anti-migration and invasion properties of 1^4+^ and 2^4+^. Although it should be noted that treatment also led to the formation of fluid-filled vacuoles in the epidermis characteristic of spongiosis, which is consistent with some toxicity effects in healthy tissue (Fig. 7).

## Conclusions

Taken together these results show that both 1^4+^ and 2^4+^ strongly disrupt actin filaments, stress fibers, and focal adhesions in highly migratory human melanoma cells. Consequently, the complexes are cytotostatic and profoundly antimigratory in 2-D cultures. More significantly, in 3-D cultures they totally inhibit the ability of an aggressive melanoma to invade the dermis of our tissue-engineered skin model, therefore meeting one of the biggest challenges for the treatment of this pernicious, hard-to-treat cancer. Given these unprecedented results, and the fact that these complexes are neither classically genotoxic nor phototoxic, but disrupt protein–protein interactions within melanoma cells, this study offers a new paradigm for the use of metallodrugs in the treatment of one of the most fatal and therapeutically intractable forms of cancer.

The observation that both complexes have this effect on melanoma but no detectable effect on migration rates of HDF cells is striking. This may just be a function of the higher metabolic rates of melanoma cells,^86^ however previous studies in a range of cell types have demonstrated that whilst the uptake of 1^4+^ is through an active, energy dependent, 2^4+^ is hardly taken up at all.^42^

The fact that both 1^4+^ and 2^4+^ are active in melanoma suggests uptake is modulated in these cells. Certainly, it is known that endocytosis, and rates of endocytosis (specifically micropinocytosis) are greatly modulated in melanoma cells^87,88^ and it is also known that 1^4+^ is internalized into cells if it is delivered through an endocytosis-compatible carrier system.^89^

The results on the tissue-engineered model reveal that that the complexes can penetrate the epidermis but do produce some deleterious effects on epidermal cells. However, if these complexes are used as a topical treatment, it is likely any off-target effects would largely be localized to the site of treatment. Given that 1^4+^ and 2^4+^ are cytostatic and anti-invasive, they could provide one component of a novel therapy. For example, although 1^4+^ and 2^4+^ are not in themselves phototoxic,^42,43^ we have developed close structural analogues that are highly photo-oxidizing, thus displaying cell killing effects even within hypoxic regions of tumor models. A topically applied combined regime of these two components would provide an anti-invasive and a (photo-)cytotoxic therapeutic as a targeted therapy for melanoma with reduced off-target effects compared to systemic dosing. Indeed, given the close structural similarities between 1^4+^ and 2^4+^ and their photo-oxidizing analogues, it seems likely that both of these functions could be provided by a single molecular architecture – studies to identify/develop such a multimodal system are already underway and will form the basis of a future report.

## Conflicts of interest

There are no conflicts to declare.

## Supplementary Material

MD-014-D2MD00280A-s001
