# Supplementary material for: Selectively inhibiting malignant melanoma migration and invasion in an engineered skin model using actin-targeting dinuclear RuII-complexes
Source: RSC Med Chem. 2022 Nov 1;14(1):65–73. doi: 10.1039/d2md00280a (PMC9890726; doi:10.1039/d2md00280a)
Supplement: MD-014-D2MD00280A-s001 [file MD-014-D2MD00280A-s001.pdf]

Electronic Supplementary Material (ESI) for RSC Medicinal Chemistry.
This journal is © The Royal Society of Chemistry 2022

Supporting Information

Selectively inhibiting malignant melanoma migration and invasion in an engineered skin model using actin-targeting dinuclear Ru^{II}-complexes.

Ahtasham Raza,[a] Stuart A. Archer,[b] Jim A. Thomas[b]*Sheila MacNeil[a]*, and John W. Haycock[a]*

[a]Materials Science & Engineering, University of Sheffield, Mappin St, Sheffield S1 3JD, UK.

[b]Department of Chemistry, University of Sheffield, Brook Hill, Sheffield, S3 7HF, UK.

Table of Contents

Material and Methods

MM 1 Materials and reagents
MM 2 Human melanoma and fibroblast cell culture
MM 3 Melanoma migration assessment using scratch migration assay
MM 4 Reversible effect of the drug on actin filaments
MM 5 Analysis of focal adhesion
MM 6 The effect of Ru(II) compounds on cellular metabolism (MTT assay)
MM 7 Melanoma spheroid formation for assessment of metabolic activity
MM 8 Collagen invasion model to study migration rate
MM 9 Tissue engineered human skin/melanoma model for assessment of melanoma migration

Additional Figures

Figure Si 1 Human (A) C8161 (B) A375-SM melanoma cell migration in serum free media
Figure Si 2. Morphology of stress fibers in untreated human melanoma C8161 (A) and A375-Sm cells that were fixed and labelled with Phalloidin (TRITC).
Figure SI 3 Effect of actin filaments after treating with $\mathbf{1}^{4+}$ for 1, 3, 6 and 24 hours.
Figure SI 4 Recovery of actin filaments after 1 hour treatment with $\mathbf{1}^{4+}$ or $\mathbf{2}^{4+}$ in 24 hours.
Figure SI 5 Focal adhesion response in human melanoma C8161 cells.
Figure SI 6 Human melanoma spheroid growth and metabolic analysis after $\mathbf{1}^{4+}$ or $\mathbf{2}^{4+}$ treatment.
Figure SI 7 Effect of $\mathbf{1}^{4+}$ on normal human tissue engineered skin model.

Material and Methods

MM 1: Materials and reagents

Ru(II) compounds (**1**$^{4+}$ and **2**$^{4+}$) were synthesized and described as previously reported (Ref 42 and references therein in main paper). Eagle's modified essential media (EMEM), Dulbecco's modified Eagles medium (DMEM), Roswell Park Memorial Institute medium (RPMI), EDTA (0.02%), penicillin, streptomycin, L-glutamine and Amphotericin B (Fungizone®), Triton X-100, Phalloidin (TRITC), Vinculin rabbit monoclonal antibody, Alexa fluor 488 goat anti-rabbit, Bovine serum albumin were all from Sigma-Alrich (Poole, Dorset, UK) and used as per manufacturer's instructions, unless otherwise stated. Fetal calf serum (FCS) was from GlobePharm Ltd, UK.

MM 2: Human melanoma and fibroblast cell culture

The A375-SM human melanoma cell lines was isolated from a lymph node metastasis of a 54-year-old female melanoma patient (developed by Professor M. J. Humphries, University Manchester, UK). While C8161 human cell line was isolated from an abdominal wall metastasis from a recurrent malignant melanoma menopausal woman (developed by Professor F. Meyskens UC Irvine (USA) via Dr. M. Edwards (University Glasgow, UK)). A375-SM and C8161 human melanoma cells were grown in melanoma culture medium consisted of EMEM media (Sigma-Aldrich) supplemented with FSC (10%v/v), L-gulamine (2uM), Penicillin (100U/mL), streptomycin (100ug/mL) and Amphotericin(0.625ug/mL) [12]. Human fibroblasts were isolated from the dermal part of split-thickness skin grafts (STSGs). Local ethics committee (Sheffield NHS trust, Sheffield, UK) approved the procedure and tissue storage and handling under Human Tissue Authority (HTA) license no 12179. Fibroblast were isolated and cultured in DMEM. All cell lines were initially grown until 80% confluent in T75 flask before detaching, counting, adjusting to required cell number and seeding.

MM 3: Melanoma migration assessment using scratch migration assay

Oris™ cell stopper technology (Platypus) was used to quantify melanoma cell movement. Briefly, the Oris system was inserted in 96 well plate as described by the manufacture's guideline. Human melanoma C8161 A375-SM and Human dermal fibroblast were seeded at an initial seeding density of $1.2x10^5$ cell/mL (i.e. each 100µL of cell suspension contained 12,000 cells). While human melanoma HBL were seeded at an initial seeding density of $2x10^5$ cells/mL (i.e. each 100µL cell suspended in a well contain 20,000 cells). The cells were incubated for 24 hours to achieve a confluent monolayer of~90%. The cells were then treated with or without1^{4+} or 2^{4+} for 1hour at 100µM (in serum free media). After 1 hour, the Oris cell stopper was removed, and cells were washed with PBS and replenished with fresh media (with 10% FCS). The cell exclusion zone was imaged at 0, 3, 6, 8 and 24hours using contrast microscopy (each variable, n = 5 wells). The microscope was connected to a camera operated by Motic Images Plus 2.0 software. Images were processed to calculate the distance and rate of migration using ImageJ.

MM 4: Reversible effect of the drug on actin filaments

Human melanoma A375-SM and C8161 cell lines were seeded on 6 well plate with an initial seeding density of $2x10^5$ cells/mL and incubated for 24 hours at 37°C at 5%CO_2. After 24 hours, cells were starved by removing existing media and replenished with fresh media with only 1%FCS and incubated overnight. This process synchronizes the cell cycle and helps to produce

consistent imaging data. The cells were then treated with or without $\mathbf{1}^{4+}$ or $\mathbf{2}^{4+}$ for 1, 3, 6 and 24 hours at 100µM (in serum free media). In another experiment, cells after 1-hour treatment ($\mathbf{1}^{4+}$ or $\mathbf{2}^{4+}$) were washed and replenished with fresh media (with FCS) and fixed after 1, 3, 6 and 24hours. After each endpoint, cells were washed with PBS and fixed with 3.7% formaldehyde (30 minutes). The cells were washed and then permeabilized with 0.1% Triton X-100 for 30 minutes. Phalloidin-tetramethylrhodamine B isothiocynamate (TRITC) was used for immuno-fluorescent staining of F-actin filaments (10µg/mL, 60 minutes). The images were taken by Ziess confocal microscopy λex=543nm; λem= 565-615nm, using Achroplan (water dipping objective 40X, NA 0.75, WD 2.1), laser power at 30%, pixel size 2048x2048, average scan speed 5, and pinhole aperture set at 1 airy unit. Three images were taken from independent areas for measurement of corrected total fluorescence intensity (ImageJ) and total actin-filament length (using NeuronJ, ImageJ plugin) in each cell (n=5 cells) and average data was compared.

MM4: Analysis of focal adhesion

Vinculin is a focal adhesion integrin-type receptor that is attached to the ECM and intracellular associated proteins. It is involved in cell adhesion and migration. We performed immuno-fluorescence analysis of vinculin on 70% confluent human melanoma cell lines (A375-SM and C8161). Briefly, the cells were treated with or without 1^{4+} or 2^{4+} for an hour or 24 hours and fixed with 3.7 % paraformaldehyde for 15min, permeabilized with 0.25% Triton™ X-100 for 10min followed by blocking by 5% BSA for an hour at RT. The cells were incubated for 3 hours at RT with vinculin rabbit monoclonal antibody at 1µg/mL in 1% BSA. Later cells were labelled with Alexa Fluro 488 goat anti-rabbit IgG secondary antibody at 1:400 (5ug/mL) for 1 hour. The images were taken using Zeiss confocal microscopy λex=488nm; λem=500-530, using Achroplan (40X), laser power 30%, pixel size 512x512, average scan speed 5, and pin hole at 1 airy unit. Three independent areas were scanned. The images were used to count the number of vinculin spots using ImageJ. The average data was plotted and compared.

MM 5: The effect of Ru(II) compounds on cellular metabolism (MTT assay)

Melanoma cell lines (HBL, A375-SM and C8161) were seeded in 96 well plates. Human melanoma C8161 and A375-SM were seeded at an initial seeding density of 1.2×10^4 cells/well while HBL was seeded at an initial seeding density of 2×10^4 cells/well and incubated for 24 hours. Cell cultures were treated with different concentrations from 0 to 200 µM of 1^{4+} or 2^{4+} for 24-hours. After incubation, the solutions were removed and MTT (thiazolyl blue tetrazolium bromide, at 0.5mg/mL in serum free media) solution was added for 2 hours. The formazan product was eluted using acidified isopropanol (30-minutes incubation at room temperature). The absorbance at 540 nm was measured using a plate reader (reference point 620 nm). The average absorbance from each concentration was obtained and the metabolic survival rate was calculated using the equation below. A graph was then plotted between survival rate and concentration.

Survival rate = (mean absorbance - baseline (no cells) / (positive control (cells no material) - baseline (no cells) x 100

MM 6: Melanoma spheroid formation for assessment of metabolic activity

3D melanoma spheroids were formed using C8161 human melanoma cell lines cultured using a liquid overly method. An initial cell seeding density of 12,000 were cultured in a 96-well plate coated with agarose gel (Type V, 1.5% w/v in EMEM) for 3 days (at 37°C and 5% CO_2).

Day 3 cultured melanoma spheroid was used to assess the growth and metabolic activity after 1^{4+} or 2^{4+} treatment. Day 3 spheroid were cultivated in a 96 well plate and treated with $\mathbf{1}^{4+}$ or $\mathbf{2}^{4+}$ (twice on day 0 and day 3) at different concentration of 10, 50, 100, 200 and 500 µM in serum free media (SFM). Growth rate was measured by calculating the spheroid size over 7 days.

On day 7, spheroid metabolic activity (viability) was assessed using MTT assay. Metabolic activity was calculated as the percentage viability relative to the untreated control spheroid (n = 6).

MM 7: Collagen invasion model to study migration rate

A three-dimension (3D) *in vitro* migration assay was used as a preclinical tool for assessing the effect of Ru (II) complexes on ECM incorporated human melanoma. The model was adopted from Charoen's published paper (27). Briefly, the spheroids were developed by liquid overlay culture method for three days. Day 3 melanoma spheroid was cultivated and then embedded in a 96 well plate containing un-polymerised collagen gel (1mg/mL Rat tail collagen 1 (Gibco)). The un-polymerised collagen plate containing the spheroids were incubated at 37°C and 5% CO_2 for an hour to allow self- polymerisation into gel (day 0). 100µL of EMEM media (with serum) was added on the on top of gel and incubated for 24 hours. After 24 hours (day1), the media was removed and replaced with $\mathbf{1}^{4+}$ or $\mathbf{2}^{4+}$ in serum free media (SFM). Melanoma cell migration from the primary (spheroid) area were imaged every day for 3 days (n =3). The migration rate was measured by calculating the area migrated by the cells (µm^2) and the number of cells presented in that area (using ImageJ).

MM 8: Tissue engineered human skin/melanoma model for assessment of melanoma migration

The in vitro tissue engineered human melanoma skin model was adopted from previously described by MacNeil's laboratory (26). Briefly, melanoma model was developed using isolated primary human keratinocytes (300K cells/model), fibroblasts (100K cells/ model) and C8161 melanoma spheroids (4-5 spheroid cultured for 3 day) co-cultured on an acellular skin dermis (de-epidermized dermis, DED) for 2 days. After 2 days of media submerged culture the model was raised to air-liquid interface culture (ALI) condition for 14 days.

While normal skin model was developed in similar method as described above except C8161 melanoma spheroids were not cultured within the model. Both normal and melanoma models were routinely fed with fresh media basolateral. After 14 days of ALI culture the models were treated with or without $\mathbf{1}^{4+}$ or $\mathbf{2}^{4+}$ or left untreated for 24 hours (n = 3). Following 24 hours dosing, the tissues were washed (PBS x3) and fixed using 3.7% (w/v) formaldehyde and processed for histology.

The primary cells (keratinocytes and fibroblast) and DED used to develop tissue engineered human skin/melanoma model was obtained from the split skin graft from consented patient during abdominoplasty /breast reduction from surgery unit (Sheffield). The procedure was approved by local ethics committee (Sheffield NHS trust, Sheffield, UK.) and tissue storage and handling under Human Tissue Authority (HTA, licence no: 12179).

MM 9: Statistical Analysis

Multiple t tests (unpaired) were performed between non treated and treated groups using GraphPad Prism software. Each group was compared using the Holm-Sidak method with statistically significant value of p ≤

Additional Figures

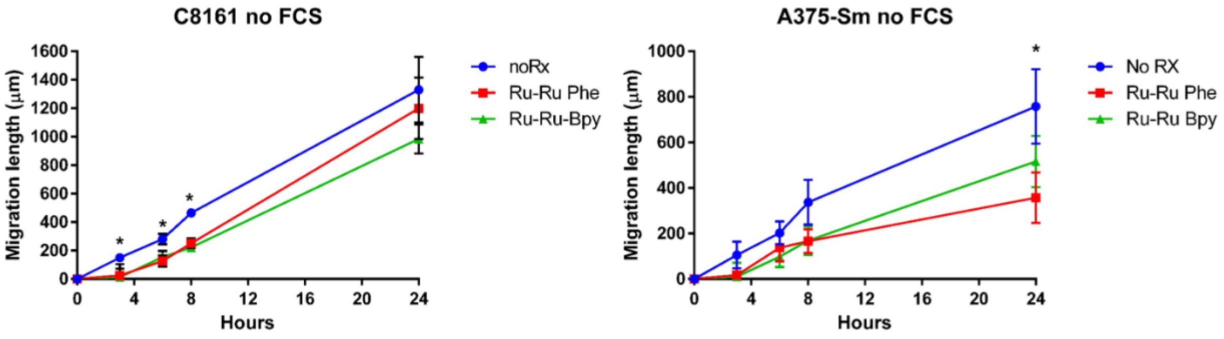

Figure SI 1.: Human (A) C8161 (B) A375-SM melanoma cell migration in No FCS media. Investigation of melanoma cell lines migration using cell exclusion zone created by cell stopper and cultured for 3,6,8 and 24 hours. Inhibition of migratory distance by melanoma after Ru (II) treatment for 1 hour. A decrease of~10 and 25 % was noticed in C8161 melanoma cell line by Ru-Ru Phe and Bpy respectively (after 24 hours). Similarly, Ru-Ru Bpy treatment in melanoma C8161 reduces cell migration to 23% as compared to 52% reduction by Ru-Ru Phe compound.

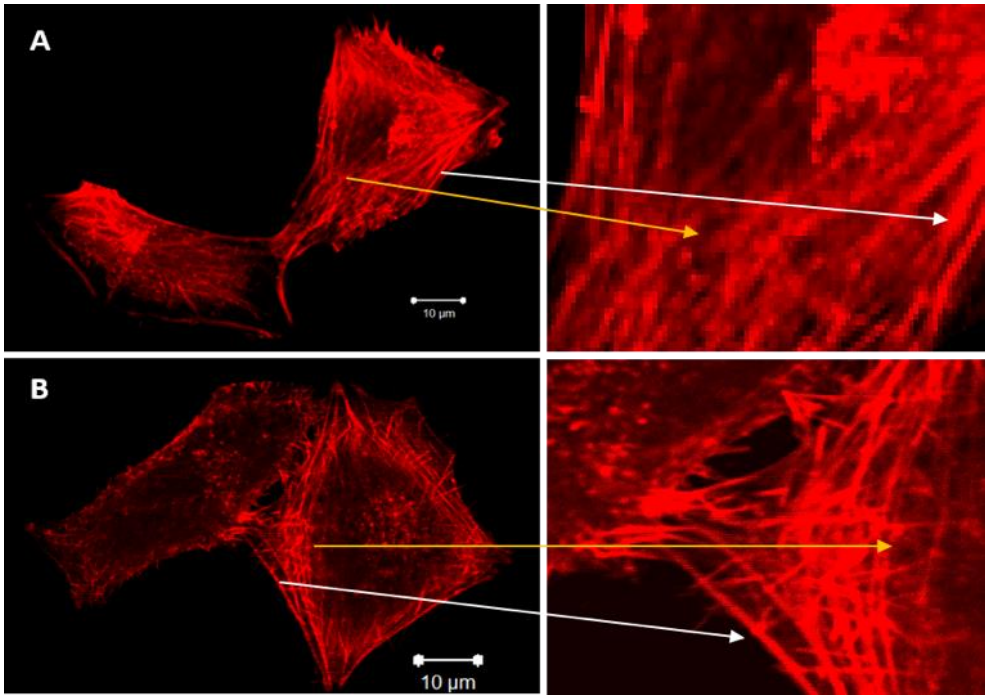

Figure SI 2. Morphology of stress fibers in untreated human melanoma C8161 (A) and A375-Sm cells that were fixed and labelled with Phalloidin (TRITC). Observe the thin central actin

fibers in the middle of cells (yellow arrow) and peripheral thick actin fibers at outer areas of cells (white arrow). Magnification 40X, SB=10 µm.

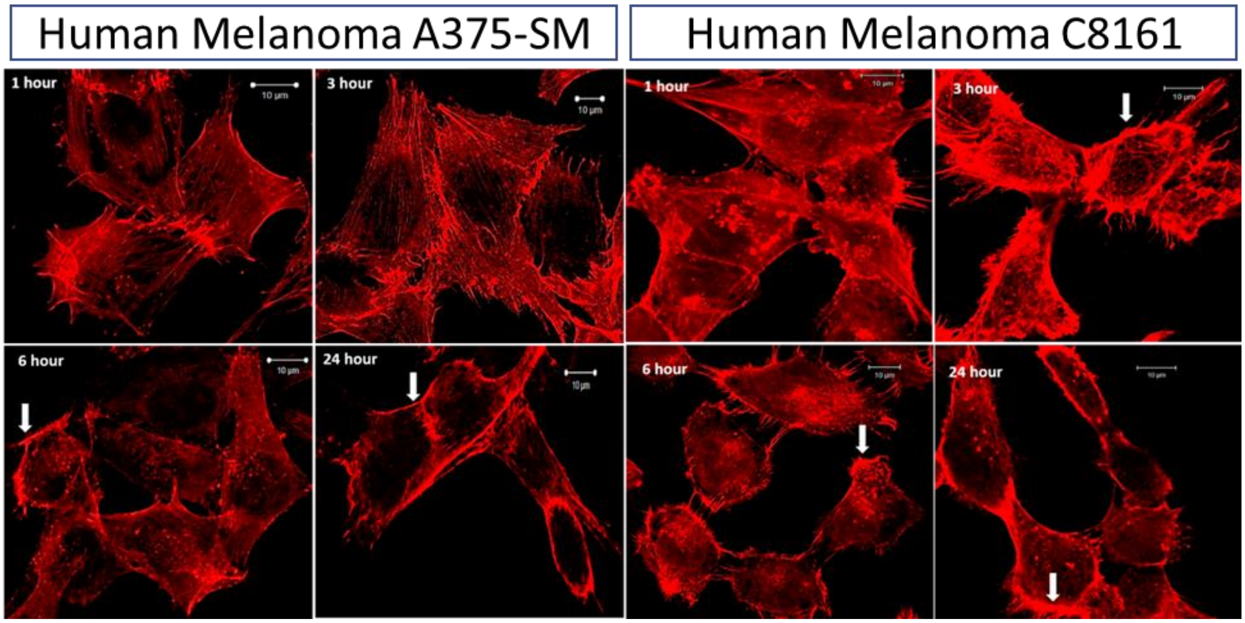

Figure SI 3: Effect of actin filaments after treating with **1**$^{4+}$ for 1, 3, 6 and 24 hours**.**
Progressive loss of central actin filaments while the peripheral filaments (arrow head) remains prominently in contact. A loss of cell size after 24-hour treatment is also visible (magnification 40X, SB 10µm).

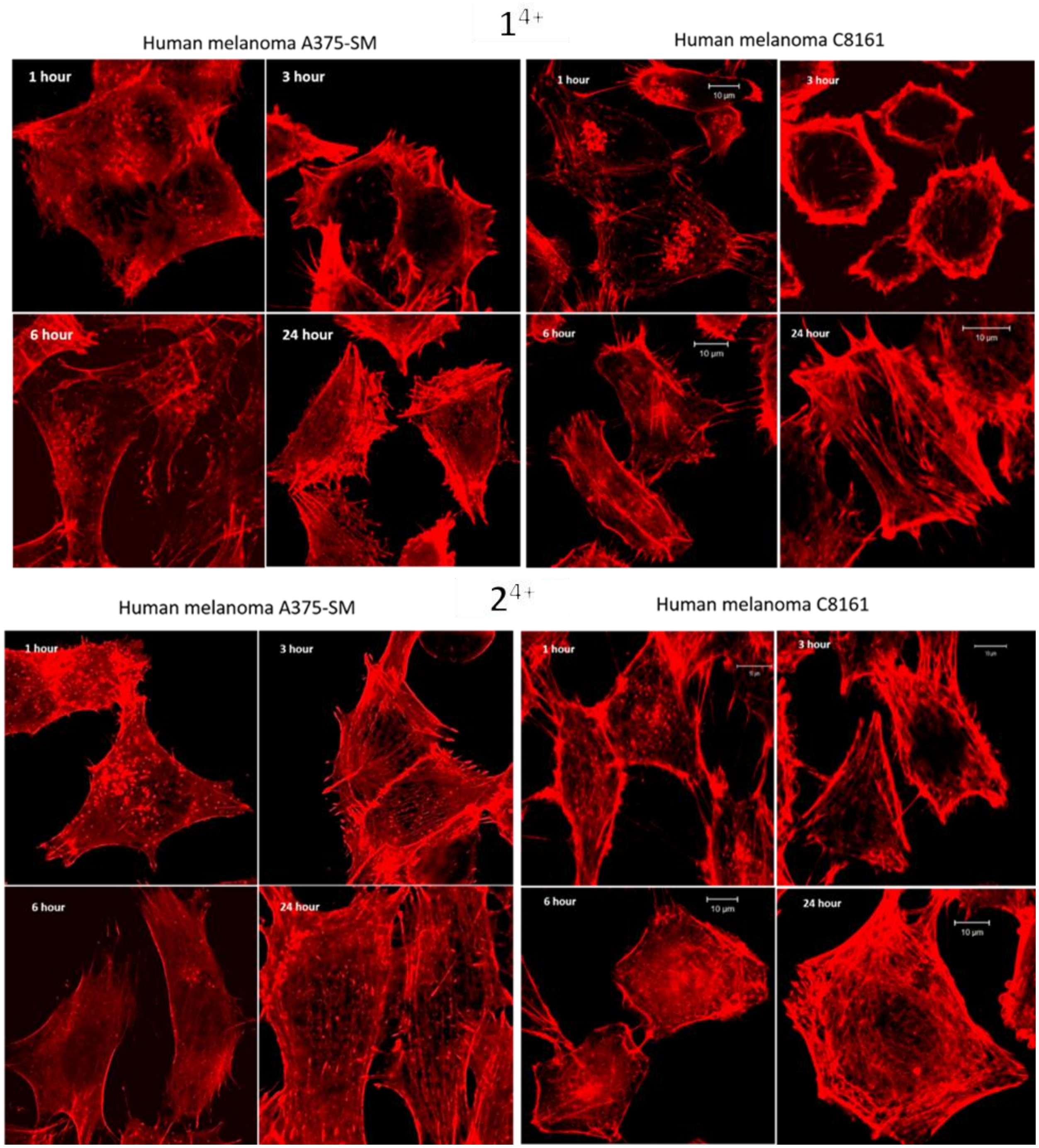

Figure SI 4: Recovery of actin filaments after 1 hour treatment with $\mathbf{1}^{4+}$ or $\mathbf{2}^{4+}$ in 24 hours. After 1-hour treatment with $\mathbf{1}^{4+}$ or $\mathbf{2}^{4+}$ (100µM) then washed and replenished with fresh media. The cells were incubated for 3, 6, and 24 hours. At the end of timepoint, the cells were washed, fixed and stained for phalloidin. The effect on actin filaments seems to remain until 6 hours. While at 24-hours, actin filaments in both cell types appears reorganized and recovered (magnification 40X, SB 10µm).

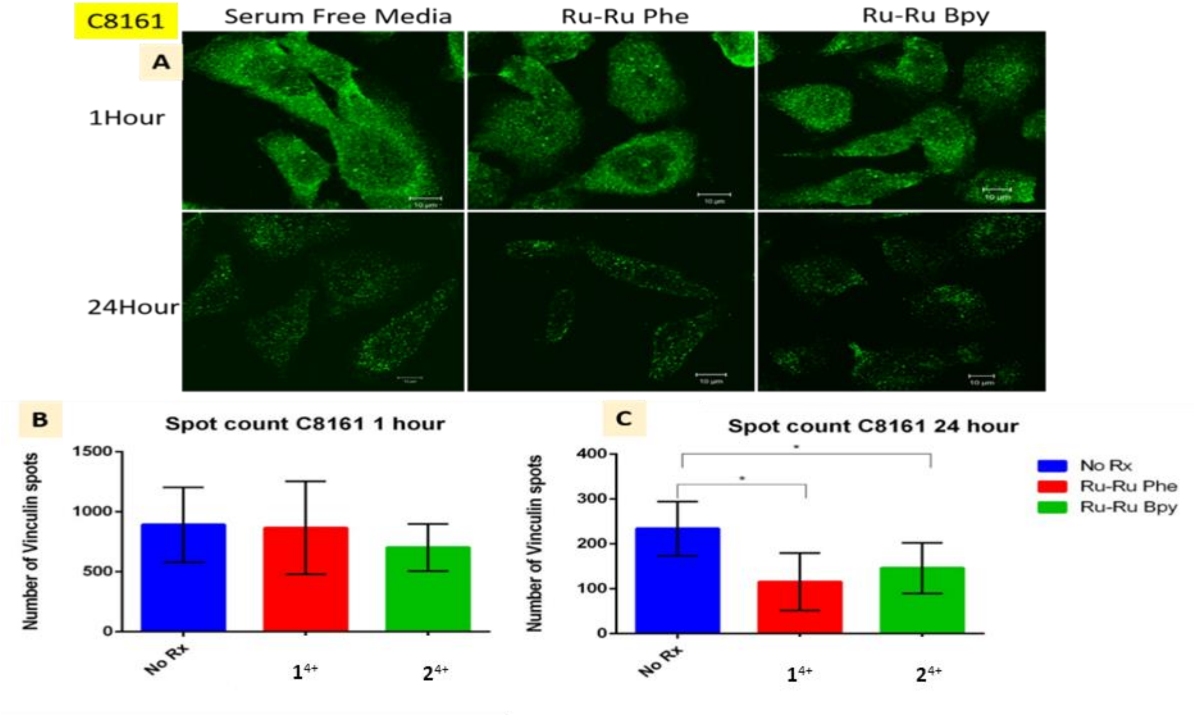

Figure SI 5: Focal adhesion response in human melanoma C8161 cells.
After 1 and 24-hour treatment with Ru(II) compounds. (A) Immunofluorescence imaging of vinculin spots within cells. (B-C) Vinculin spot count after (B) 1 hour and (C) 24-hour treatment (p= 0.05, significant difference between no treatment and **1**$^{4+}$ or **2**$^{4+}$ was 0.04 and 0.01 respectively).

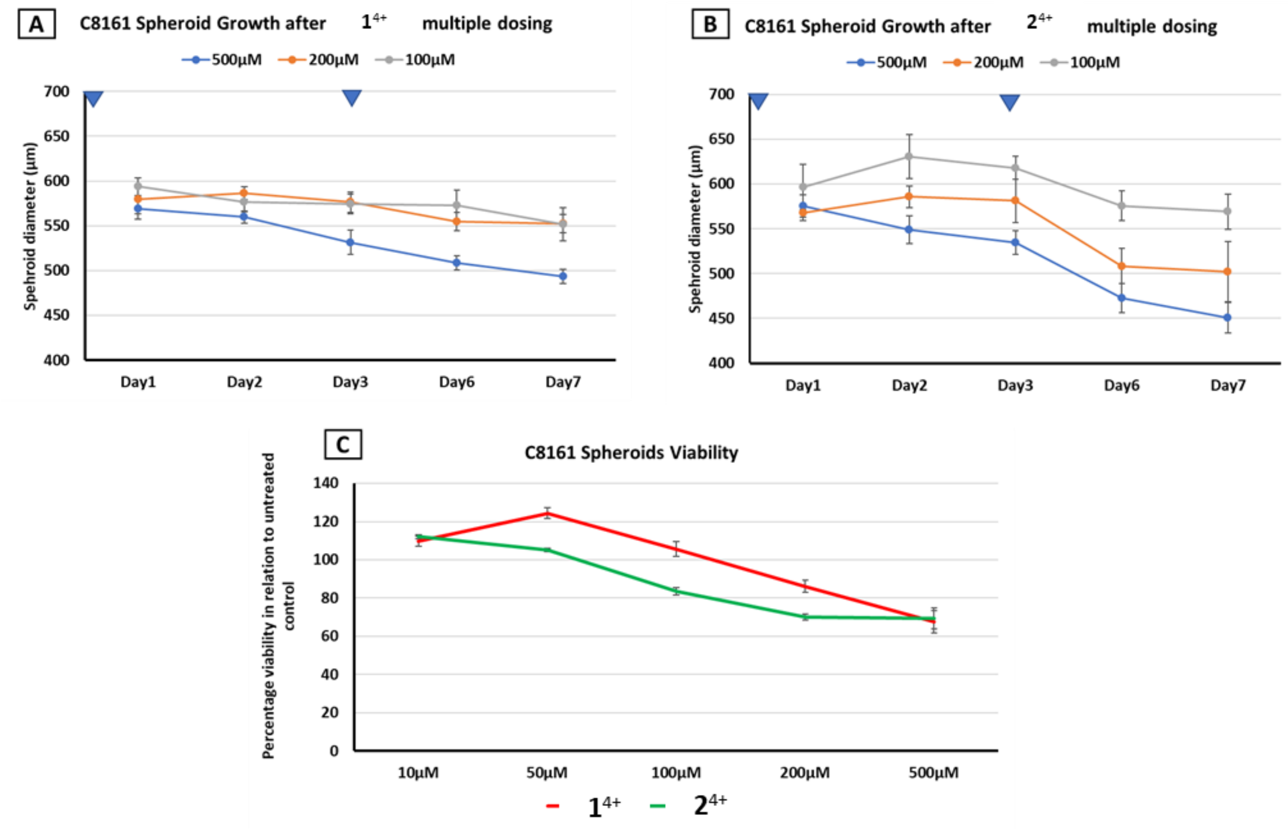

Figure SI 6: Human melanoma spheroid (A-B) growth and (C) metabolic analysis after **1**$^{4+}$ or **2**$^{4+}$ treatment. Spheroid's diameter was measured after treating on (▼) day 0 and day 3 with (A) **1**$^{4+}$ (B) or **2**$^{4+}$ at different concentrations (10, 50, 100, 200 and 500 µM) over 7 days. Growth curve was generated by calculating the spheroid diameter (mean ± SEM µm) plotted over 7 days (n=6). (C) Spheroid viability was measured at day 7 using MTT assay. Metabolic activity was calculated as the percentage viability relative to the vehicle (serum free media) control spheroid (mean ± SEM µm, n = 6).

---Internal Use---

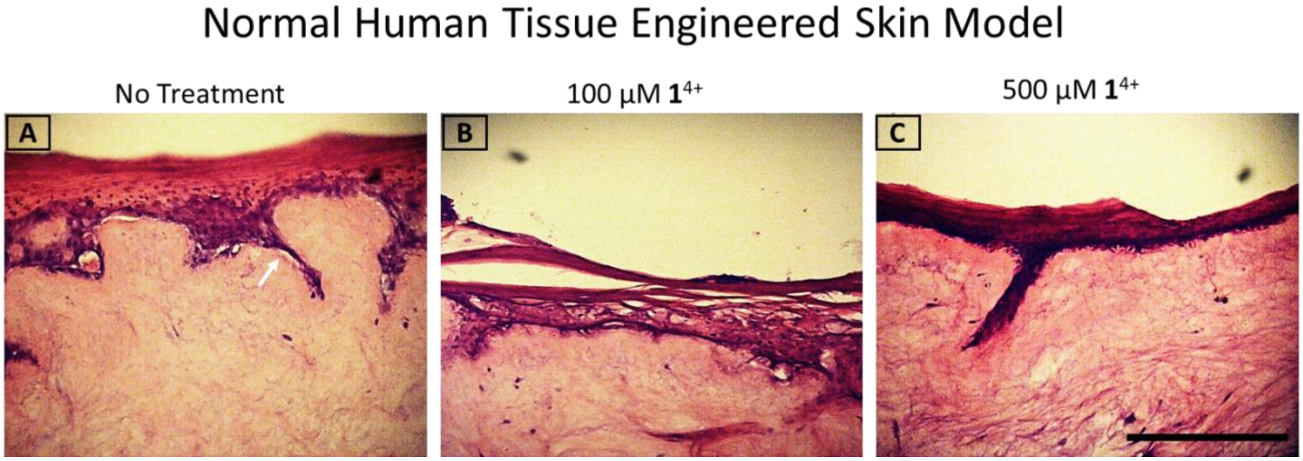

Figure SI 7: Effect of $\mathbf{1}^{4+}$ on normal human tissue engineered skin model.
The model was developed using primary cells (human isolated fibroblast (100,000) and keratinocytes (300,000) cultured together on a human De-epithelialized dermis for 14 days (air-liquid interface culture conditions). The model was dosed with $\mathbf{1}^{4+}$ at (B) 100µM, (C) 500µM or left untreated (control, A) for 24 hours. Observe normal (A) tissue consisting of multi-cellular layer with a top keratinized top layer, it has the features of a normal human skin like rete ridges (white arrow) and no invading cells in the dermis. In treated group (B-C) the dose dependent toxicity was noticed. (B) The separation of the epidermal and dermal layers was noticed due to disintegration of the dermo-epidermal junction (basement membrane) at low concentration. (C) The thickening and sloughing of the stratum corneum (Parakeratosis) were noticed uniformly across the whole sample at higher concentration (Magnification 20X, SB = 500 µm).
